# Analysis of Representations of the Aid That Public Psychological Support Points Provide to Adolescent Female Victims of Gender-Based Violence: Reformulation of Policies and Practices

**DOI:** 10.3390/ijerph19148422

**Published:** 2022-07-10

**Authors:** Isabel Cuadrado-Gordillo, Guadalupe Martín-Mora-Parra, Ismael Puig-Amores

**Affiliations:** Faculty of Education and Psychology, Department of Psychology and Anthropology, University of Extremadura, 06071 Badajoz, Spain; guadammp@gmail.com (G.M.-M.-P.); ipuigamores@unex.es (I.P.-A.)

**Keywords:** gender-based violence, adolescents, qualitative study, psychological intervention

## Abstract

Gender-based violence in adolescence has become a social health problem that is creating great concern and interest worldwide. In this regard, knowledge of the role taken by the professionals responsible for screening, detecting, referring, and caring for adolescent victims is essential to be able to understand the phenomenon and its characteristics in a practical way. In this sense, psychologists specialising in the care of victims of adolescent gender-based violence have complete and in-depth knowledge, not only of the phenomenon itself and the way in which it is presented in society, but also of the features presented by these victims and the aspects that need to be worked on during the intervention process. Given this context, a series of interviews with psychologists specialised in the care of gender-based violence victims were subjected to a qualitative deductive/inductive analysis. These interviews addressed the psychologists’ theoretical-practical knowledge about the adolescent gender-based violence phenomenon. The analysis of the results points to the victims’ irrational ideas regarding abusive relationships, to the form in which Psychological Support Points are organised to help the victims, and to the strengths, weaknesses, and needs of these centres for them to be able to improve their operation and effectiveness in providing comprehensive care for adolescents beyond the psychological consultations themselves.

## 1. Introduction

Gender-based violence in adolescence is a phenomenon that affects young people worldwide, and its prevalence is even greater than in adult couples [1,2].

The heterogeneity of this phenomenon, and the number of variables involved at its onset and in its maintenance, have complicated its approach from both the point of view of prevention and that of detection and intervention. The difficulty is compounded considering that the victim-survivors themselves are often unaware of the situation of victimisation, so that they are unlikely to seek help. For example, Spanish government data reveal that one in three females do not identify behaviours such as control as being abusive [3]. Similarly, various abusive behaviours that would not be accepted under normal circumstances are tolerated and naturalised under the prism of romantic love [4]. In this regard, according to the National Institute of Statistics of Spain (INE) the total number of survivor-victims of gender-based violence is greater than the number of people reported, with a ratio of 1.6 survivor-victims for each person reported [5].

Considering this phenomenon, health professionals (doctors, nurses, and psychologists) have an essential role to play in the care of victims of gender-based violence. Doctors and nurses are at the frontline of psychological care within the public health system. They play a fundamental part in both the screening and referral of adolescent victim-survivors for their intervention process, as well as for the guidance and help to those victim-survivors’ families [6]. In this sense, various research studies have pointed out the strengths and weaknesses observed in Spain’s public health system with respect to the psychological care provided to victims of gender-based violence, as well as the needs detected [7,8].

The role of psychologists, as experts in mental and emotional health, is equally fundamental. Victim-survivors of adolescent dating violence tend to experience a large number of internalising-type psychological symptoms (depression, anxiety, hostility, etc.), as well as a lack of adequate resources and coping skills to overcome such situations [9]. Similarly, these victim-survivors exhibit a large number of externalising symptoms. Specifically, the presence in the victims of risky behaviours such as the consumption of substances such as alcohol and marijuana has been highlighted [10,11], and this is ultimately predictive of the phenomenon of polyvictimisation [12,13]. Likewise, the consumption of alcohol or drugs seems on occasion to become a maladaptive resource to cope with the situation experienced [14,15], further aggravating the situation.

Taking into account the above considerations, it is absolutely essential for a victim-survivor to receive psychological treatment, not only to face the psychological consequences deriving from victimisation, but also to strengthen their personal and emotional skills to be able to prevent it in the future. Despite this, there have been few studies that have analysed the role of psychologists and the specialised centres where they carry out their professional work, which, more than primary care, would attend and care for adolescent victim-survivors of gender-based violence who have been referred from other health services.

Within the Spanish public health system, the mental health services are responsible for attending to any patient suffering from psychological and/or emotional problems. However, these centres do not have such a high operating efficiency as primary care centres, mainly due to the waiting lists that cause consultations to be delayed. In this sense, there has been a warning that in cities such as Madrid, the waiting lists for psychological care for minors entail a delay of up to five months [16].

Taking into account the above context, the various Spanish regional governments have promoted the creation of additional resources dedicated exclusively to the care of female victims of gender-based violence. These resources, which have the objective of bringing attention to this serious social health problem, usually depend on institutions other than the National Health System.

In Extremadura (Spain), the Psychological Support Points (henceforth PAPs, from their denomination in Spanish—Puntos de Atención Psicológica) are units framed in the Attention to Victim-Survivors of Gender Violence Network and developed within the actions undertaken by the Regional Board of Equality and Cooperation for Development.

These units were set up more than a decade ago with the purpose of offering advice, diagnosis, and intervention to female victims of gender-based violence who come to these centres voluntarily, or are referred from some other health, community, or educational centre [17]. With this, the target users of these units include not only adult females, but also their minor-age sons and daughters, as well as female adolescents who are victimised in their romantic relationships.

Since their foundation, PAPs have steadily been gaining in importance, as is reflected in the numbers of patients attended. Focusing attention on female adolescent patients who are victim-survivors of gender-based violence, one can observe a clear evolution reflecting the growing presence of this phenomenon in society. Thus, in 2016, these resources assisted 16 adolescents in Extremadura who were victim-survivors of gender-based violence, as well as 1 victim of sexual abuse [18]. These numbers grew in 2018 to 19 adolescent victim-survivors of gender-based violence being treated and 15 adolescent victim-survivors of sexual abuse [19]. Recently, the 2021 data revealed that the number of minor-age victim-survivors of gender-based violence assisted in Extremadura PAPs had grown to a total of 30 [20]. In contrast, the resources allocated to care for victim-survivors of gender-based violence do not seem to be increasing at the same rate.

In this context, and taking into account all the problems found, the main purpose of this study was to analyse a series of interviews with psychologists from the PAPs in the main cities of Extremadura (Spain) about adolescent dating violence. The objective of these interviews was to determine and integrate for analysis the vision and knowledge that psychologists have about victimisation in adolescent gender-based violence. Specifically, the intention was to determine:

- Organisational aspects of the PAPs, the resources they have, and the strengths and needs of these public attention and intervention centres for victims of gender-based violence.
- The knowledge that the psychologists have about the characteristics of victim-survivors, the most prevalent types of violence among the adolescents who come to the PAPs, the maladaptive problems and ideas that these victim-survivors present, and the needs that have to be addressed during the intervention process.

Subsequently, the questions asked during the interviews were divided for analysis into three different categories that integrate all the aspects addressed in a complete and comprehensive manner. The present research is, to the best of our knowledge, the first qualitative study carried out in Spain exploring the phenomenon of adolescent dating violence from the perspective of the psychologists who are currently working in assisting the victims.

## 2. Materials and Methods

This study was designed on the basis of the criteria established by COREQ [21] for reporting and publishing qualitative studies. This method is based on a 32-item list that guides the design, the collection of information, the description of the research sample, and the analysis of the data, among other aspects.

Likewise, this research was carried out within the framework of a broader project developed in three different phases (“Indicators…”). The project aims to assess and analyse gender-based violence in adolescent couples in a broad manner, taking into account the perspectives of the victim-survivors (Phase 1), of the primary care doctors who are in a privileged position to detect cases of violence (Phase 2), and of the psychologists at the PAPs, specialised in interventions with the said victim-survivors (Phase 3). The first phase reveals the characteristics that teen dating violence has in actual society and how factors such as sexism, moral disengagement, negative emotions, or the use of substances such as marijuana can affect victimisation [11,22,23,24]. The second phase reveals the strengths, weaknesses, and needs that primary care doctors present in the diagnosis and attention to the particularities of each woman, and also provides some solutions identified by the physicians themselves [7]. This last phase intends to offer complementary information that can be added to the rest of the results and conclusions revealed in the previous phases of the project.

The PAPs are integrated into the network created by the Junta (Regional Government) of Extremadura to provide comprehensive services in matters of gender-based violence, including specialised care for the victim-survivors and their children, and for minor-age victim-survivors of said violence. The functions of the PAPs focus on the prevention, detection, intervention, and awareness of gender-based violence. This study belongs to the third phase of the study: Identification of the strengths, weaknesses, and needs of the PAPs focused on support for victims of gender-based violence.

### 2.1. Participants

The participants included in this study were a total of 11 psychologists. These psychologists were working in the 10 PAPs in the most populated areas of Extremadura (Spain)—5 in the province of Badajoz, and 5 in the province of Cáceres. These units are located in cities of different population sizes and belong to different areas within the Region (both urban with more than 50,000 inhabitants, and rural with fewer than 50,000 inhabitants). With this, the sample covers all the socioeconomic and cultural characteristics present in Extremadura. Additionally, the authors went to the Casa de la Mujer (the Women’s Centre) in Badajoz. The participants’ profile is shown in the following table (Table 1).

All the psychologists showed great interest in the study, and participated in the research voluntarily once the objectives pursued and the way the research had been carried out in the previous phases had been explained. All the psychologists interviewed were female. The PAP staff comprises almost exclusively women psychologists. These psychologists had extensive experience in the field and involved themselves in the study showing great motivation.

### 2.2. Procedure

Prior to the beginning of this third phase of the Research Project, authorisation was obtained from the general director of the Instituto de la Mujer of Extremadura, as well as from the Board of Equality and Cooperation for Development of the Junta de Extremadura. Once obtained, authorisation for the interview was requested from the management of each of the PAPs. Likewise, legal advice and approval was requested from the Bioethics Committee of the University of Extremadura (Spain) (Ref. 18/2017).

Once these authorisations had been obtained, the ten PAPs were contacted by telephone. In this first contact, the objectives of the study, content of the interviews, and the approximate duration were explained. Likewise, the anonymity of all participants was guaranteed.

The interviews began in 2018 and continued throughout 2019. All the interviews were carried out ad hoc by the principal investigator of the research project, who personally visited all the PAPs after making an appointment with the psychologists. All the interviews were conducted in a quiet environment, inside the psychologists’ office within the PAP, and ensuring that there was enough time to answer and discuss the questions (approximately 90 min). Each interview conducted was recorded in an audio file, after obtaining the consent of the participants. Likewise, they were all transcribed for analysis. This transcription was performed by one of the project researchers.

### 2.3. Interview

The interview was semistructured, prepared in the form of a guide, following the objectives set in the research, but allowing for some flexibility in the questions.

To this end, the research team determined the format and type of questions to be used, their wording, sequence, and order. Likewise, the topics to be addressed were identified, taking into account the results obtained in the first and second phases of the research. The first phase had focused on the collection and analysis of data from 2577 adolescents between the ages of 14 and 18. This analysis allowed the researchers to obtain a deep understanding of the phenomenon of adolescent dating violence, its characteristics, problems, and risk indicators. The second phase of the research included the deductive/inductive thematic analysis of interviews conducted with 95 primary care doctors from the public health system of Extremadura. This analysis provided an in-depth view of the strengths, weaknesses, and needs of the Extremadura public health system in relation to the detection and care of the victims of gender-based violence in adolescent dating relationships. The results of both previous phases of the research, as well as an extensive review of the scientific literature, were taken as the basis for the preparation of the interviews and the experiences lived daily at these PAPs for victimised females, these being essential elements of knowledge for the psychologists.

The interview guide contained 12 open-ended questions, divided into three different categories that included distinct key questions. These questions were aimed at obtaining an in-depth view of the organisation, coordination, intervention, monitoring, and main problems faced by the professionals working in the PAPs. The following table (Table 2) presents the main themes and key questions that formed the complete guide of the interviews carried out.

To ensure the understandability and efficiency of the questions, these were sent to experts in clinical psychology to thus maximise the value of the interview.

### 2.4. Data Analysis

The transcription of all the interviews was carried out by one of the psychologists belonging to the research group in charge of the project, who was already familiar with the objectives of the study and the results obtained in the previous phases of the research. These transcripts were read and reviewed by all the team members to familiarise themselves with the data and discover the internal structure to be analysed.

After familiarisation with the data, the analysis of the transcripts was performed by means of the method of structured content analysis, a method widely used and described in various studies [25,26,27]. Additionally, deductive/inductive thematic content analysis [28] was used as a referent, which was aimed at identifying, finding, and analysing the common underlying patterns in the different interviews carried out with the goal of defining, naming, and elaborating the final report. Thus, the first phase of analysis was developed through a deductive analysis, creating an initial coding system based on the main themes and questions included in the interview guide (see Table 1). These codes had to be interpreted by the researchers who then assigned them labels based on the information the psychologist expressed. Once the codes had been applied, the information was summarised in short sentences. For example, an explanation that provided details about different aspects of the abusive relationship was classified as characteristic of gender-based violence in teen dating.

Next, the second phase of the analysis continued by performing an inductive analysis of the transcripts, with the goal of detecting subcategories and analysis codes within the said subcategories. This step again required the interpretation on the part of the researchers in order to establish the relationship between the previously established discrete inductive codes. With this, the classification of the codes was carried out based on their actual content, as well as depending on the guide provided by the questions on which the interviews were based. In this way, the greatest possible number of subcategories and codes were identified. In this sense, for example, a participant explained when answering the question of problems detected in adolescent victim-survivors how these victim-survivors have incorrect thoughts about their relationship; this answer was classified as distortions and maladjusted beliefs instead of characteristic of victim-survivors.

In both phases of the analysis, the research team involved in the project met on several occasions to discuss and compare the categories and themes extracted, as well as the selected text passages in relation to those themes. During the process of analysis, the initial code list was progressively redefined. These codes were finally organised into topics and subtopics. Additionally, to analyse the data objectively, summaries of the responses obtained in the interviews were created through the construction of a table-grid that additionally included the percentages of the different responses provided by the experts. Finally, at a second moment, the preliminary work carried out was reviewed again to guarantee the validity and reliability in the coding and interpretation of the data, making various contributions and clarifications.

## 3. Results

With respect to the research questions, the following categories were deduced: (a) Organisation of the PAPs, (b) Victim-survivors, (c) Intervention. Each category was divided into subcategories that cover different topics or thematic content, as can be seen in Table 3.

**Figure 1:**
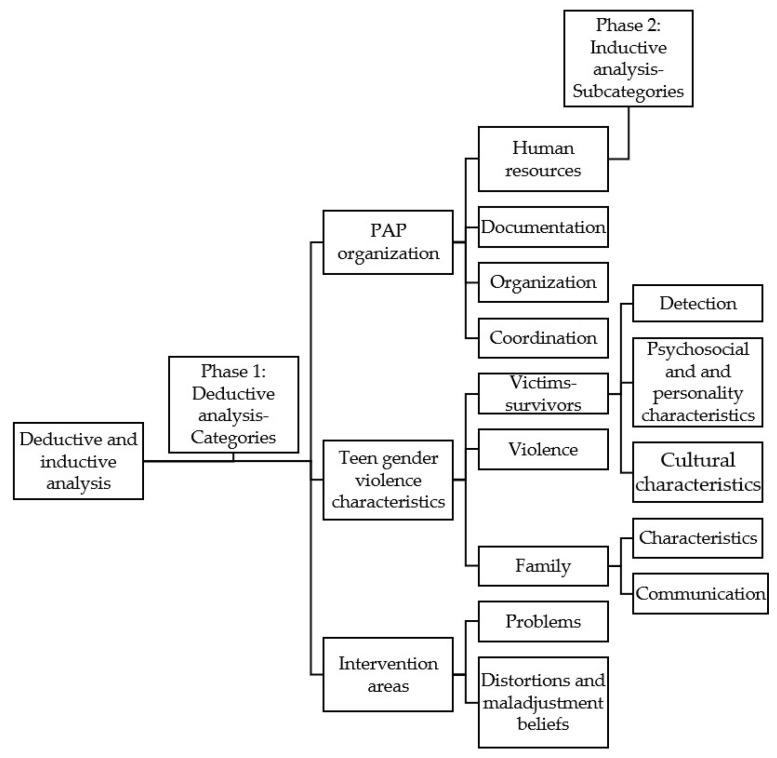
Research phases and categories.

Finally, some of the subcategories identified were divided during the analysis into distinct codes that allow different aspects, although related, to be differentiated within that subcategory. The different phases followed during the analysis (deductive and inductive analysis), as well as the categories, subcategories, and codes established, are shown in Figure 1. A summary table (Table 4) is added that includes the main answers and the frequency of the responses that the psychologist gave to these.

### 3.1. Category 1: Organisation of the PAPs

This first category deals with the way in which the PAPs are organised. Different aspects were analysed, such as the psychologists themselves who are part of these care teams for battered women and how they come to form part of them, the documentation that is available to them, and how they organise themselves to participate in prevention programs at the same time as they carry out their intervention tasks as psychologists with the victims, or the protocols used to coordinate the actions of the different professionals who come into contact with the victims of gender abuse. In this way, four subcategories were established: (1) Human resources, (2) Documentation, (3) Organisation, (4) Coordination.

#### 3.1.1. Subcategory 1: Human Resources

Employment status and type of contract that the psychologists who work at the PAPs in Extremadura have.

Most of the psychologists interviewed explain that unlike in the case of primary care doctors, the employment situation of PAP psychologists is highly precarious. Far from depending on the Board of Health and Social Services of the Junta de Extremadura, they usually depend on the Town Councils of the cities where they are located. These councils hire, via competitive civil service examination (“opposition”), one psychologist every year, sometimes leaving the service without responsible professionals for several months, and therefore without any kind of attention for battered women.

This way of organising the PAPs poses obvious problems for the psychologists. In addition to the continual job insecurity they suffer, the fact that the professional responsible for the PAP is constantly changing implies an added difficulty when it comes to creating the climate of trust and intimacy necessary for the victims to tell, honestly and without fear, the reason why they have come. Additionally, the fact that for several months of the year there is no psychologist in the centre also prevents many women and adolescents from going directly to the resource, which is another very marked difference with respect to the way in which the primary care centres in the Extremadura health system work. The latter, due to their solidity and permanence over time, encourage patients to feel that their doctors “know” all the members of the family perfectly, and to be able to turn to them for any problem or doubt about their health. On the other hand, most of the patients come to the PAPs referred from other areas. In this way, one of the psychologists interviewed revealed:

“Of course, but because I’ve been here for two years. Before there was another companion. Well two years … less. It’s that job insecurity also… I mean, I started in 2018, in June, until December. In December you get fired. I had to present myself again to the “opposition” exam, I entered again in June and it is until December. In other words, here there are 6–7 months when this stays empty”.

#### 3.1.2. Subcategory 2: Documentation

The documentation and data that the PAPs of Extremadura make use of to know the data of prevalence and evolution in relation to the phenomenon of gender-based violence.

During the interviews, questions were specifically asked about the annual prevalence statistics in order to analyse how many adolescent victim-survivors received treatment and help, as well as the evolution of the treatment and the phenomenon itself over time. In this sense, however, it was surprising to find that half of the psychologists interviewed explained that they did not have any data of this type, despite the fact that the said data are collected, stored, and shared through the pertinent reports. In this way, one of the psychologists interviewed indicated:

“No, because we don’t even have data within the women’s institute itself, which is something that strikes me, because we do send the data, but then there is no website where we can enter and see the data. Just like we send that data developed by age, by… we don’t have it”.

Likewise, another psychologist added:

“We do not have these data. I assume they are being collected somewhere. I don’t know”.

With this, a large number of these professionals are unaware of the cases that occur annually throughout the region, or the way in which the phenomenon has evolved over time.

#### 3.1.3. Subcategory 3: Organisation

The way the PAPs collaborate with other entities to detect cases of gender victimisation in the adolescent population. Likewise, aspects such as the participation of the psychologists of the PAPs in different prevention programs, while they continue with their intention work in the units, were also addressed.

Beginning with the detection of the victims, the psychologists interviewed explained that most of the victim-survivors came to the PAP by referral from other areas. Thus, the detections were not carried out by the psychologists themselves, but other people from the victim-survivors’ environment were the ones who did this, thereby carrying out an essential task. In this regard, one of the psychologists belonging to these PAP units revealed:

“No, no detection here. That is, the PAP as a PAP, no. The office of equality, yes” … “Apart from the fact that this point is not included in the intervention protocols of the PAPs. It is collected in what is the intervention with the women”.

With this, the commonest places where victimisation is detected, according to the professionals interviewed, are the Oficinas de la Mujer (the Women’s Affairs Offices), secondary schools, public primary health care centres, and in the most serious cases, the police or the courts. With this, collaboration and coordination between these areas and the PAPs becomes essential.

“Many times, in the courts, when there is already an accusation and such, they already give them all the information, which includes our services. This includes the home, so we call, many times they come. Thus, the path can begin from any environment. From the health field sometimes too. Referrals from health centres also because they sometimes have a case there”.

#### 3.1.4. Subcategory 4: Coordination

Documents that the PAPs use to guide and coordinate their interventions with the rest of the organisations and professionals involved in the prevention, detection, and intervention of gender-based violence in adolescents.

In this regard, all the psychologists interviewed pointed out that there is no type of document, protocol, or official body that establishes or regulates the collaboration between institutions:

“There is no protocol that establishes a coordination after referral, okay?”

Nonetheless, some of the psychologists pointed out that despite the nonexistence of any official coordinating protocol, there is the resource of the so-called “territorial roundtables” (local and territorial). These “roundtables” are meetings that are held periodically which involve bringing together professionals from different fields to share information and coordinate their actions (police, teachers, psychologists, doctors, etc.). They therefore have great theoretical potential for the prevention and intervention of gender-based violence in adolescence. However, it is noted that most of the professionals invited usually do not attend, so that these meetings do not manage to fulfil the objectives they had when they began to be held. Thus, P9 explained:

“There are coordination roundtables at local level, and then at territorial level, where the social, educational, and health services are supposed to go… whatever. The health workers never come… and neither do other professionals”.

### 3.2. Category 2: Characteristics of Gender-Based Violence in Adolescent Dating

This category corresponds to an analysis of the characteristics presented by the victim-survivors of adolescent gender-based violence who attend the PAPs from the perspective of the psychologists responsible for these units. These professionals have first-hand experience that allows them to build a broad and detailed vision of what these victim-survivors are like, and thus are able to point out not only their general characteristics, but also contextual factors, and indicators that may be of great importance in the prevention of this phenomenon. In this regard, the analysis detects three different subcategories: (1) Victim-survivors; (2) Commonest types of violence; (3) Family.

#### 3.2.1. Subcategory 1: Victim-Survivors

The profiles and main characteristics of the victim-survivors who attend consultations at the PAPs to be treated, taking into account psychological, social, cultural, and economic characteristics.

Thus, this subcategory is divided into the following analysis codes: detection; psychosocial and personality characteristics; cultural characteristics.

##### Detection

A large proportion of the psychologists interviewed indicated that the detection of victim-survivors is not carried out at the PAPs, and that they usually come derived from other areas. Thus, it is common to find that educators, when situations of violence are detected, recommend the victims or their families to go to the PAP for an appropriate evaluation and intervention. Likewise, it is also relatively common for mental health centres, given the saturation of the mental health service, to refer victims of gender-based violence to PAPs to receive faster care. Thus, in this regard P2 noted:

“Primary Care doctors perhaps refer more often to the Mental Health service. Indeed, to Mental Health. What happens is that Mental Health then often refers to us. Of course, because the waiting time in Mental Health is two months, even three months or three and a half months, and, of course, here that doesn’t happen”.

Likewise, P1 pointed out that usually the victims come to these PAPs following the advice of their own family, school, friends, etc., and they rarely come on their own initiative. In this sense, collaboration with the school and the family can improve the comprehensive care and the satisfaction of these psychologists with their professional work.

##### Psychosocial and Personality Characteristics

Most of the PAP psychologists interviewed pointed out that there is no exact psychosocial profile that can be generalised to all the adolescent victim-survivors who attend their consultations. This means that the adolescents who are cared for in the PAPs come from diverse sociocultural and economic backgrounds. This fact is reflected in the response given by P8:

“Gender-based violence is occurring in all social spheres, at all economic levels, in all types of education, in professions of all types, and the same continues to occur”.

In general, this same situation of diversity is reflected in the search for a specific profile regarding the personality characteristics of victims. Thus, the participating psychologists explained that it is impossible to establish a personality model associated with gender victimisation in adolescent couples, as they had found a great variety and heterogeneity of their psychological traits.

##### Cultural Characteristics

Regarding the influence of culture, some psychologists believed that certain ethnic groups, such as the Roma or Arabs, have customs and social values that abet victimisation, with this aspect therefore linked to the characteristics of the families from which the victims come. Belonging to a “traditional” family encourages the adolescents to behave with their partners in a similar way as their parents do at home, and this behaviour is also supported by the parents themselves. More specifically, P5 revealed:

“What we, that is, the Spanish population do not approve of is behaviour that the Moroccan population thinks is normal”.

Additionally, the psychologists opined that it is extremely difficult to access these victims of gender-based violence, since when any type of action is carried out to approach the adolescents or their families, these actions tend to be rejected due to the prejudices that these cultures usually have towards psychology.

#### 3.2.2. Subcategory 2: Violence

The commonest types of violence suffered by adolescent victim-survivors who attend consultations at the PAPs.

In this regard, all the psychologists who participated in the study pointed out that psychological violence is predominant in adolescent dating relationships. Within psychological violence, there are various types, the commonest being emotional abuse, blackmail, insults, control, etc.

Nonetheless, all the psychologists who attend victim-survivors at these PAPs pointed out that, although the adolescent aggressors rarely seem to resort to physical violence, both this type of violence and sexual violence are also present in these cases, often hidden under the halo of normalisation that makes the victims unable to recognise this type of abuse until reaching more advanced phases of therapy. Thus, aggressors who force victim-survivors to have unprotected sexual relations, or pushing and pulling, tend to be seen as showing forms of normal behaviour that, on many occasions, are interpreted as being forms or manifestations of love when they are accepted. In this sense, P11 pointed out:

“It is psychological violence, and sexual violence. In adolescents, sexual violence is very common… To say, if you don’t sleep with me I am going to leave you. If you don’t sleep with me I leave you. So, you will see”.

#### 3.2.3. Subcategory 3: Family

The commonest types of families that go to consultations at the PAPs. This category analyses the types of communication and collaboration that is established with families in the PAPs when their daughters begin a psychological intervention process after victimisation has been detected.

This subcategory is divided into two different analysis codes: characteristics of families, and communication.

##### Characteristics of the Families

Most of the psychologists from the PAPs indicated that the families are completely different from each other, and that they, the psychologists, are unable to establish a clear pattern of any family nucleus associated with adolescent victimisation. Therefore, aspects such as the socioeconomic or family cultural status of the adolescents who attend the units are varied. Nonetheless, the participants also noted that the characteristics the victim-survivors present and the way the parents relate to each other are vital, not only to detect victimisation but also to detect how the victim-survivors relate to their partners. Thus, the conservative families in which gender stereotypes and sexism (hostile or benevolent) are predominant seem to be strongly associated with the girls having a similar role in their dating relationships. Likewise, it is especially noted how many mothers seem to tolerate this type of behaviour due to the normalisation of violent processes, which causes adolescents not only to assume these values as their own, but also fosters the perpetuation of victimisation. In this regard, P8 noted:

“If at home there has been, for example, this type of relationship, it is more difficult for them to leave. It is also a factor that we have. If her parents, for example, have a healthy relationship, it is easier for her to realize that her relationship is not like her parents’, and there she can make a… But of course, if your relationship is exactly the same as your parents in an environment of abuse and such, because it will be much more difficult to get out of there, of course”.

##### Communication

Communication is a key element in the assistance provided to adolescent victim-survivors of gender-based violence from a dual perspective. On the one hand, communication is revealed as being essential in the processes of prevention, care, and protection of the victim-survivors. Thus, the psychologists interviewed revealed that communication with the families is not problematic. In most cases, the adolescents go to the PAP consultation when the situation is already serious, so the families are highly motivated to follow the instructions of the professionals who are helping them. Nonetheless, the psychologists interviewed pointed out that the interaction with the parents is minimal, being reduced to the first contact and familiarisation interviews, to subsequently generate an environment of trust with the victim-survivor. During this stage, the adolescents are the protagonists. In this way, it is possible to build a relationship of trust in which the young victim-survivors understand that, only in specific cases related to sensitive issues that entail some type of danger for them and that are dealt with within the consultation, would their parents be informed. Thus, P3pointed out that:

“If I believe that an issue needs to be discussed with her mother, I tell the adolescent that I want to discuss it with her mother. Later, there are things that I discuss with the mother, especially the mothers, without telling the adolescent, but I never go into intimate things that she has told me”.

And on the other hand, the psychologists explain that it is necessary to communicate with the parents to prevent them from blaming their daughters. Some families seem to hold the victim-survivors themselves to be responsible for the situation they are experiencing, especially when the adolescents resume the relationship with their aggressors after having broken up with them for a while. Thus, a more serious situation ends up being generated, since the girls may even hide the situation they are experiencing in order to avoid feeling judged by their parents. In this regard, P2 indicated:

“But I, if there are times that, with the parents, especially if I see that it is causing her discomfort because… they tell you about it in therapy. I don’t know, as at home they blame her a lot for that. Or imagine that she has gone back with him, or whatever. So, if it makes her uncomfortable, there are times that I talk to the families to explain to them what the process of gender-based violence is, what the cycle of gender-based violence is, and explain how they can help”.

### 3.3. Category 3: Areas of Intervention

The third category analyses the aspects in which the psychologists must intervene during consultations with adolescent victim-survivors within the PAPs. In this category, two different subcategories were found: (1) Problems, (2) Distortions and maladjusted beliefs.

#### 3.3.1. Subcategory 1: Problems

During the interviews, most of the psychologists who participated in the research coincided in pointing out that all the victim-survivors present a series of common difficulties. These problems are directly related to the way in which adolescent girls conceive of love and relationships. In this sense, the idealisation of love that popular culture promotes through different representations (novels, films, television series, music, etc.) causes young girls not to understand the reality of relationships. In this sense, certain behaviours such as jealousy, control, obsession with the partner, etc. are given a romantic character. All these aspects, which are experienced by the victim-survivors as manifestations of interest and love, lead to situations of psychological and emotional abuse from which it is difficult to escape due to the lack of awareness that adolescent girls have about these situations. In this regard, P4 indicated:

“They do not detect the situation because, now more than ever, I see the difference with my generation. Now there is like an exacerbation of the myths of romantic love. And there is an easiness in the control. For example, you talk to the adolescents, right? When they are already in consultation, right? And they come for a few reasons, either a physical attack... or... that is, something more serious. When you start talking to them about behaviour of… emotional abuse or psychological abuse, at first, they don’t identify it because they think that’s part of the relationship. In other words, jealousy, control, which is very easy now, right? With social networks, with WhatsApp, you know? So, of course, it’s like… it’s difficult, once they come in, you can already do a restructuring, but at first they see it as if that’s normal”.

#### 3.3.2. Subcategory 2: Distortions and Maladjusted Beliefs

In relation to the distortions and maladjusted beliefs that adolescent victim-survivors usually present, all the psychologists interviewed specified that the adolescent girls who come to their consultations share a series of beliefs related to female empowerment and false equality. This false empowerment and sexism disguised under the paradigm of benevolent sexism are causing gender prejudice to be more present than ever among some adolescents. In this way, P5 commented:

“And many times they are girls who believe they are more empowered, or the false empowerment. That showing themselves that being more sexualized, that all these things on the social networks, is a big risk factor… they believe they are more empowered, and they suffer more relationships of harassment”.

Thus, many of the victimised-survivor adolescents feel that within their relationships, they have the same power that their partners have over them. Therefore, to show said power and position they have regarding boys, they themselves become aggressors, exercising the same type of role, trying to copy the behaviour that their partners have with them. In this sense, P7 indicated:

“The problem now is that many of them say that they are the same as… that is, it is not that they believe that they are to blame for the situation, but that they have similar behaviour. So, you talk to them about control, and they tell you, but I also control him. Because I’m jealous too, you know? It is as if it has been equated… they think that they do have an equality in their relationship because they have behaviour that is similar to what he has with her”.

## 4. Discussion

This study has explored, from the point of view of psychologists, the phenomenon of gender-based violence in adolescent dating. To this end, a series of interviews were conducted that were recorded in audio format and later transcribed to be analysed following a deductive/inductive type of analysis. The analysis of the results provides relevant data not only about the phenomenon of gender victimisation in adolescent dating, but also allows one to observe the positive and negative points that the PAPs have. In this sense, the study contributes to understanding the strengths, weaknesses, and needs that exist within the field of specialised psychological care for adolescent victim-survivors of gender-based violence, as well as the characteristics, errors of thinking, and main aspects on which to intervene during the therapy process. Additionally, the results found complemented the previous phases of our research developed in teen dating violence, providing accurate information to design prevention programs with detailed information adjusted to the characteristics and current reality of adolescents.

### 4.1. PAP Organisation

Starting with the organisational structure of the PAPs, the analysis of the results brings out the job instability to which these units are subjected at the level of human resources. In contrast to what is the case with medical professionals who work in the area of primary care where stability is one of the strengths, the situation of the psychologists specialised in caring for victims of gender-based violence at the PAPs is more unstable, with the selection process being repeated annually. This situation additionally implies that the unit remains closed during this period of time, thus causing a delay in the interventions, which even come to a standstill for several months. With this, the provision of greater human and financial resources to the PAPs is essential to be able to optimise the effectiveness of these units [29].

The first direct consequence entailed by this way of organising human resources affects the victim-survivors of gender-based violence. Thus, neither the primary care doctors from the public health service nor the victim-survivors themselves can count on the psychological assistance resource that is offered by the PAPs for long periods of time, which leaves going to the mental health service of the public health system as the only alternative. This area also has its own problems, since in many cases, its units are overwhelmed, making quick attention impossible [16,30]. Likewise, and additionally, the fact that these PAPs do not offer their psychological care services throughout the year makes it difficult for the potential users to know and recognise this resource. With this, the detection and subsequent care of potential victim-survivors is further complicated. In this sense, one of the main strengths of the Spanish public health system is the application of a primary health care model that promotes the stability of health professionals, and with this, the assurance of psychological care for the users. In this way, the doctors and nurses care for the same patients over long periods of time, specifically from adolescence to the last stages of people’s adult lives. This fact allows the relationship of trust between the doctor and patient to be strengthened, thereby encouraging the users to go to their Primary Health Care provider when they need to [8]. In this sense, transferring to the PAPs this model and the positive aspects that the Public Health Care system presents could represent a great advance in the care of victims of gender-based violence.

Likewise, another finding regarding the functional organisational network of the PAPs for victimised-survivor females highlights the lack of coordination with other local resources that are equally specialised and related to the intervention respecting female victim-survivors of violence, as well as with other professionals of different fields in the same territorial zone in which the different PAP units operate and where the events took place. With regard to the availability of technical resources that help to provide correct attention or intervention, the psychologists interviewed declared that they do not have an official protocol that specifies the steps that must be adopted when faced with an adolescent female who is a victim-survivor of gender-based violence. It is noted that this finding is not an isolated fact, and has been pointed out in other studies carried out in different areas of Spain. Thus, it was found the same type of problems in the Principality of Asturias (Spain), revealing the existence of organisational barriers that make it difficult to care for victimised-survivor females [31]. Those authors note that aspects such as the variability among registries, the lack of inter-institutional accessibility to them, or even their simple absence, constitute a major problem that greatly complicates the effective care of the victims. Gallego and Torrejón [32], in their recent study carried out in the Region of Murcia (Spain) about the care for the victims of gender-based violence, reveal that among all the possible needs detected, the area with the most margin for improvement is precisely the coordination among the resources that assist the users, with this aspect being pointed to by more than 30% of their sample.

The psychologists interviewed made clear some attempts to mitigate the effects of the organisational deficiencies found. Thus, the participants referred to the intention of linking the different professionals involved in the detection and intervention of victimised females through the organisation of recurrent meetings or “territorial roundtables”. The need for this type of connection had already been noted as being vital in previous studies. For example, a study that analysed a total of 2503 questionnaires responded to by health staff from 24 hospitals revealed the need for a more dynamic administration, as well as the need to improve coordination between the different units and services so as to increase the quality of care for patients [33]. Other study [34] pointed out the importance of properly managing the resources that are available in the care of victim-survivors of gender-based violence, especially highlighting the need to coordinate the sectors of education, social services, legal attention, police, etc. Nonetheless, in this regard, the territorial roundtables do not seem to be effective as an attempt to connect and exchange data. Thus, the psychologists interviewed revealed that certain professionals, such as educators, doctors, police, etc., do not attend these exchanges of experiences, possibly because some of them do not know about them or lack the information that informs about the holding of these meetings, while others subordinate their assistance to their work responsibilities. These criticisms were also pointed out by the medical professionals from the primary care centres of the public health system. In this way, they highlight as a fundamental aspect of intervention the need to coordinate all the resources, both material and human, with the aim of providing a comprehensive, specialised, and multidisciplinary intervention for victims of adolescent gender-based violence [7,35].

Bearing in mind that the phenomenon of violence in adolescent dating is heterogeneous and brings various professionals together in its prevention and detection, referral, and care, the disconnection found between the various resources that provide care to victim-survivors does not seem to be effective or functional. Thus, the absence of contact could even be the reason why the psychologists who work in these specialised units rarely participate in the prevention programs that are initiated in educational centres, for example. The very specialisation of the PAPs would imply a certain degree of isolation, which would make them largely unknown to the general population, as well as to professionals who work in other areas related to health. Thus, one of the greatest challenges to be faced at these specialised units is not only focused on achieving adequate coordination and connection with the rest of the services involved in caring for victim-survivors, but also involves increasing the visibility of this type of care service aimed specifically at victimised-survivor females [36].

### 4.2. Teen Gender Violence Characteristics

Another relevant finding related to the care that the psychologists provide to victim-survivors of adolescent gender-based violence reveals that the psychologists who work in these specific units indicate that they do not have any special difficulties in communicating with the families. This fact facilitates care for the victim-survivors, while simplifying the establishment of a relationship of trust during therapy. In this way, this care system for victimised-survivor adolescents adopted by the PAPs brings together the advantages of autonomous models of care for patients, as well as the so-called “health coaching” models that reveal the importance of active participation in their treatments [37,38]. In this sense, the victimised-survivor adolescents become the protagonists of their own treatment, even being aware of the exceptions established to the confidentiality of the data that they may reveal during therapy.

Far from lying, the psychologists explain directly to the adolescents that in the event that situations or facts that are potentially dangerous for their own life are revealed during the consultation, the necessary actions would be put in place to prevent the said dangerous situation from being perpetrated, with communication with their families and the disclosure of the said information to their parents being included among these actions. This active communication encourages the patients to get involved in their treatment, avoiding passivity and disconnection [39]. This type of approach additionally avoids the loss of confidence that would be generated in the victims when they discover that they have been lied to in some way. This is a documented phenomenon, for example, in terminal cancer patients who discover that their doctors have been withholding relevant information relative to the course of their disease [40].

Nonetheless, the psychologists point out an exception to the few difficulties they have in their interactions with the family. Specifically, the social and cultural origin context of the adolescent patients and their families transforms the communication when it encourages the acceptance of violence and traditional gender prejudices and stereotypes. In this regard, the psychologists interviewed specifically explain how Muslim victim-survivors are probably the most difficult to care for. These young adolescents receive education and values influenced by the culture of their families in which they do not usually promote equality, but rather the opposite, submission towards males. The permanence of sexist values and traditional gender roles in these cultures reinforces discrimination and the assumption of the role of victim-survivor even in younger women [41]. In contrast, Spanish victim-survivors present different characteristics. For example, although it is true that gender stereotypes are still present in Hispanic culture, the evolution of the society and the education that young people have received has led to a new generation with fewer stereotypes, who know what gender violence is, but at the same time, consider this phenomenon to be a problem linked to adults [42,43]. Therefore, hostile sexism seems to be linked with conservative ideology [44], while benevolent sexism shows its influence in today’s society [22,45]. With this, the paradigm shift that must be made when caring for culturally different patients must go beyond overcoming language barriers [46].

It is also essential to understand that different cultural backgrounds have an influence beyond the acceptance of traditional gender roles. In this sense, misunderstandings can often arise between what the psychologist prescribes for the recovery and what the patient understands should be done. In this way, it has been noted that immigrant populations, especially the Chinese and the Moroccan, present additional resistances related to psychology and psychological treatments due to the prejudices that these cultures continue to present in face of this science and the stigma of mental illness [47,48], which prevent these potential patients from being able to enjoy a specialised and integrated care [49]. Lastly, it is also important to understand with regards to foreign victim-survivors that, on many occasions, they depend directly on their partners because of administrative reasons, even when they are young. Many of them may be in the country irregularly, and do not trust the authorities [50], a fact that leaves them defenceless before the law where the condition of migrant prevails over that of victim-survivor, especially when it is not possible to prove abuse [51].

Given the difficulties generated by the problems encountered, similarly to the rest of illnesses, the solution could require psychologists to adopt a more leisurely, empathic, and comprehensive approach [40], an aspect that, however, is not easy to achieve due to the limited human resources that the PAPs have and the time these professionals need to build a relationship of trust with the victim-survivor, as it has been pointed out in studies with different ethnicities and cultures [50]. The lack of coordination between all the professionals involved, as well as the lack of an integrated overall analysis of the situation of migrant women, and the problems with access to economic aid, make the situation of these victimised-survivor females even more difficult to address [51]. Once again, the establishment of a coordinating system that regulates the interactions between these services could be the key to resolving the problems detected in the care of foreign victims.

### 4.3. Intervention Areas

Finally, the data analysis reveals how gender prejudices and sexism play a major role in the explanation that the psychologists make of the phenomenon of adolescent dating violence. In this way, these characteristics are present in all the cases that are attended to in the PAPs’ consultations. Nonetheless, the form in which they appear is different. While in Muslim cultures the beliefs in gender roles are associated with the traditionalism and conservatism that is typical of their culture and religious beliefs, in Spanish victim-survivors this kind of stereotype is associated with completely different factors linked to the evolution of society. In this sense, false empowerment [52,53] is mentioned by most of the psychologists interviewed. This false empowerment encourages women to develop a false sense of autonomy and freedom while living in a society that continues to promote a patriarchal structure [54] in which the sexual objectification of women [55] and benevolent sexism [51,56,57,58] are more present than ever, especially among victimised-survivor adolescents [59].

Thus, the psychologists interviewed indicated that victimised-survivor adolescents in Spain arrive at consultations expressing empowerment and liberation, as well as showing great confidence in the equality of the role they play within the relationship. This fact contrasts with the psychological, emotional, and sexual violence that marks the abuse that their partners exert on them. In this way, according to authors such as [60], it would perhaps be more accurate to interpret that in today’s society, women are not being empowered, but that there is rather a neoliberal interpretation of feminism.

From the PAPs, it is explained that a large proportion of the adolescent victim-survivors develop erroneous beliefs and maladjusted behaviours that end up being reflected in the perpetration of behaviour that is similar to what they receive from their aggressors. This phenomenon had been noted previously [61], and more recently it was revealed that more than half of adolescent victim-survivors indicated that they responded to violence with violence [51], similarly to the case in the context of cyberbullying with aggressive victims [62]. In this sense, the feminist theory of Dutton and Golant notes that violent responses by the victim-survivors are on many occasions used as a defence [63]. Nonetheless, the explanations that psychologists specialising in gender-based violence provide seem to go beyond revenge or maladaptive forms of coping. Thus, abusive behaviours such as jealousy and monitoring the mobile phone or social networks have spread and have become normalised, being seen as proof of love [64]. These actions, which adolescent girls also feel the right to exercise, appear equally in both members of the couple, due to their normalisation in the context of dating relationships [65,66]. Therefore, it is possible that the normalisation of violence, in combination with false empowerment, is behind this current phenomenon of adolescent dating violence according to the psychologists.

Taking the above into account, it could be inferred that the victimised-survivor adolescents are taking as their own the values that today’s society promotes through the different manifestations of popular culture (music, cinema, youth literature, etc.) [67] and social networks [68]. Adolescents incorporate into their own value system those constructs that justify practically any behaviour or conduct in the name of romantic and ideal love [59,69]. Therefore, the naturalisation of violence goes beyond visible physical and/or psychological violence limited to specific contexts, being expressed through cultural, economic, and social patterns established by the heteropatriarchal power groups [70,71].

Thus, violence against females is of a structural character since it encompasses the entire social order. These structural differences, in turn, end up being reflected in the various forms of gender inequality. With this, each and every one of the areas of society (rules, ideology, sense of humour, religion, etc.), are oriented towards the reproduction of dominance over women [72], and the said exercise of power may be traced at all levels of real life [73]. Finally, all these forms of inequality and dominance end up reflected, at a microstructural level, in violence and abuse directed towards women [74,75]. Consequently, the formal equality that is increasingly being perceived socially by women in general and female adolescents in particular seems to be hiding a much more complex reality that masks inequality under these formal structures [76]. Much of the work of the psychologists specialised in the intervention on adolescent gender-based violence should therefore go through working on these aspects with the aim of making the hidden visible, thereby minimising the chances that the victimised adolescents end up in adulthood becoming women who are also victimised.

These findings have implications for policy and practice as they flag the weak points in the attention which adolescent victim-survivors of gender violence receive. Providing more resources (both human and material) is needed, especially considering that the teen dating phenomenon is the type of abuse that has increased the most in recent times [5], even surpassing gender violence in adult couples [1,2]. Despite the efforts that have been made to provide better psychological support to victim-survivors, the lack of connection between all the parties involved (primary care doctors, teachers, social workers, police, psychologist, families, adolescents, etc.), complicates both prevention and intervention actions. This paper also points to the need for a detailed knowledge not only about cultural differences and how these differences affect the way violence is interpreted, but also about the new forms of sexism that affect today’s society and the normalisation of violence. Incorporating these factors to formal training of the professionals involved could undoubtedly improve comprehensive care from a broader perspective.

## 5. Limitations

This study may present some methodological limitations; fundamentally, those related to the self-reported nature of the data given the difficulty of being able to verify them. Nonetheless, this subjectivity has been minimised in this study by comparing the data with other sources. In addition, having the opinions of the school psychologists and teachers who, in education centres, are in contact with the victim-survivors on a daily basis could complete the conclusions established in the present research. Finally, analysing the risk of burnout in psychologists specialised in gender violence would be another interesting aspect to study, considering that this fact could affect the quality of care provided to victim-survivors. These limitations could serve as a guide for future research to complete the body of knowledge on this topic.

## 6. Conclusions

This study has highlighted the way in which the PAP units, specialising in the treatment of gender-based violence, are organised. Likewise, the strengths and weaknesses have been analysed from the perspective felt and experienced by the psychologists who work in them. Finally, the specialised knowledge that these psychologists have contributed has allowed for a greater understanding of the structural evolution of the phenomenon of adolescent dating violence in a sociocultural context.

More specifically, one of the most relevant contributions of this study points to the limitations that specialised care for adolescent victim-survivors of gender-based violence presents. Thus, the lack of coordination among all the agents involved in the detection, referral, and care of these victim-survivors, as well as the job instability to which these professionals are on many occasions exposed, create barriers that make intervention and treatment even more difficult. Likewise, it has revealed the evolution that the phenomenon of violence in adolescent dating is tolerated. In this sense, the very evolution of society that is marking the promotion of gender equality at a formal level continues to point to differences at microstructural levels, which seems to be the cause of false empowerment. This false empowerment becomes the reason that encourages some of the victim-survivors to exercise, to a large extent, the same type of abusive behaviour that they suffer or have suffered from their romantic partners, the aggressors. The cultural silences of society, as well as the individual silences of the victim-survivors, continue to be anchored in an adverse social structure, with all these aspects being observed in the public health system, also considering the individual, family, social, cultural, and institutional factors.

From this position, the specialised intervention for victimised-survivor adolescents is insufficient, thus making it necessary to create a system that, with a multidisciplinary approach, can coordinate the linkage of all the relevant agents involved in the process. In this way, true preventive actions can be developed that promote the modification of maladjusted beliefs, while at the same time making the specialised services of attention to victim-survivors known, as well as connecting the different professionals, ultimately facilitating the detection of the potential victim-survivors.

## Figures and Tables

**Table 1:** Participants’ profile.

Participants *	Area	City/Town	Province
P1	Urban	Badajoz	Badajoz
P2	Urban	Cáceres	Cáceres
P3	Urban	Badajoz	Badajoz
P4	Urban	Don Benito-Villanueva	Badajoz
P5	Rural	Jarandilla de la Vera	Cáceres
P6	Urban	Plasencia	Cáceres
P7	Rural	Zafra	Badajoz
P8	Rural	Miajadas	Cáceres
P9–10	Rural	Olivenza	Badajoz
P11	Rural	Navalmoral de la Mata	Cáceres

* Age category 40–55.

**Table 2:** Themes and key questions included in the interview guide.

Topics	Key Questions
Organisation and coordination of PAP	How do you start working in the PAPs? Inside the Spanish public health system, which body do the PAPs depend on?
	What are the prevalence statistics of teen dating violence handled by PAPs?
	What is the victim-detection procedure followed in the PAPs?
	Are there collaboration protocols with educational or health institutes?
	How are these protocols implemented?
Profile and characteristics of the victims	What are the risk and vulnerability factors of those who are subjected to this type of violence?
	What are the psychosocial traits that characterise the victims of teen dating violence that you see in your practice?
	What are the sources of information to which adolescents turn to detect that they are in a situation of victimisation?
Areas of intervention and problems detected	Of the cases you are presented to you, what are the most common types of abuse that you must treat?
	What are the sexist stereotypes of adolescents?

**Table 3:** Analysis categories and subcategories.

Main Category	Subcategories	Topics
Organisation of PAPs	Human resources	Psychologist, type of employment contract, job access method
	Documentation	Estatistics, shared data
	Organisation	Participation in prevention programms, detection of victims
	Coordination	Protocols, collaboration permissions among professionals
Characteristics of gender-based violence in adolescent dating	Victim-survivors	Victim-survivors profile, risk factors, sociodemographic characteristics
	Types of violence	Psychological, emotional, physical,
	Families	Implication, communication, comprehension
Intervention areas in gender-based violence in adolescent dating	Problems	Sexism, gender stereotypes
	Distortions and maladjusted beliefs	False empowerment, aggressive victims

**Table 4:** Summary of answers and frequency of responses given by the psychologists.

Questions	Answers and Frequency of Responses Provided by the Pyschologists						
How do you start working in the PAPs?	Competitive civil examination (11)						
What are the prevalence statistics of teen dating violence handled by PAPs?	No data (10)			17–18% of teen dating violence (1)			
What is the victim-detection procedure followed in the PAPs?	Family (4)	Equality (3)	Primary health care (1)	Education (3)	Friends (2)	Police (3)	Mental Health (3)
	Court (1)						
Are there collaboration protocols with educational or health institutes?	There is no protocols for adolescent victim-survivors (11)			Territorial roundtables (4)			
How are these protocols implemented?	No protocols (11)						
What are the risk and vulnerability factors of those who are subjected to this type of violence?	Romantic love (8)	Normalisation of violence (8)		Patriarchal society (6)		Sexualisation (5)	
	Friends (4)	Pooverty (3)	Social inequalities (2)		Porn (5)	Family and culture (8)	
What are the psychosocial traits that characterise the victims of teen dating violence that you see in your practice?	Low self-esteem (4)		No profile (11)	Dysfunctional family (6)			
What are the sources of information to which adolescents turn to detect that they are in a situation of victimisation?	Internet (8)		Friends (6)	None (4)		Teachers (3)	
What are the most common types of abuse that you must treat?	Psychological (10)		Sexual (6)	Emotional (5)	Verbal (4)	Physical (2)	
What are the sexist stereotypes of adolescents?	Gender stereotypes (9)			False empowerment (10)			
